# Reflex Colic and Reflex Diarrhœa

**Published:** 1909-02-20

**Authors:** 


### Diseases of Children.

### REFLEX COLIC AND REFLEX DIARRHŒA.

For many reasons the name "reflex diarrhœa" is superior to the more common one of "lienteric diarrhœa." It indicates to some extent the causation of the trouble and the appropriate lines of treatment. "Lientery" is a name which has been handed down by authority, and its meaning is rarely understood by practitioners, although the name conveys a mental picture of the affection. It is derived from the Greek words λεῖος (smooth) and ἔντερον (an intestine), and is defined in the Lexicon of Medicine published by the New Sydenham Society as "a species of diarrhœa, or looseness, in which the food passes rapidly through the bowels undigested, and nearly in the same condition as it was when taken . . . . so called because the food seems to have slipped over a smooth-lined intestine."

The affection may occur at any age, but is most common in the sixth year of life. This is perhaps due to the presence of an exciting cause at this age in the shape of dentition, leading to bolting of food and imperfect digestion. It may occur even in infants, and it is by no means uncommon to find that each feeding is immediately followed by an action of the bowels. Strictly speaking, such reflex action of the bowels is not often lientery, for it is unusual to find the food passed through the whole alimentary canal so rapidly and unchanged. Nevertheless, the affection is a true reflex diarrhœa. The presence of undigested food in the stools from this cause at any age does not mean that the food has passed through the bowels from mouth to anus in the short time which elapses between ingestion and defæcation, but that it is passed out of the stomach too rapidly to be properly digested, and consequently appears in an undigested form in the stools. Many a time in infants the mother says the babe passes its food from the bowel just as it has been taken in. This is a curious fact, for it is practically certain that gastric digestion in infants is not a necessity, and that complete utilisation of the ingesta can be accomplished by the intestinal juices. The necessary proviso is that the food stays a sufficiently long time in contact with the digestive juices, and is not hurried through the gut by unduly rapid peristalsis. For it is unduly rapid peristalsis which is the cause of this peculiar diarrhœa. As soon as food enters the stomach this reflex peristalsis is set up, is followed by griping pains or colic, and compels the child, if old enough, to hurry to the closet. On examination the stools consist of undigested food and mucus.

These children are usually of the nervous type, excitable, neurotic, highly-strung, and variable in temper. Infants, too, no doubt, show over excitability of the nervous system, probably the result of the undeveloped state of the higher controlling nerve centres and the preponderance of reflex activity. The attacks at this early age are generally the result of unsuitable feeding as regards the quantity or quality of the food, sometimes due to the food being given at too high or too low a temperature, occasionally a complication or sequel of acute enteritis. Similar exciting causes may be present in older children. A common one is the habit of bolting the morning meal in a great hurry in order to be in time for school. Other factors coming into play are the hurried bodily movements after meals and the sudden exposure to change in temperature on leaving the house. In its simplest form reflex diarrhœa is set up by chill, or by the introduction of cold food or drink, notably ices, into the stomach when heated. Colicky pains may be at once induced and the bowels act profusely, with abdominal discomfort or even severe pain. Such attacks are generally easily relieved by warmth and temporary abstinence from food and drink.

Reflex colic is a less well defined condition, but there are strong reasons to suppose that it is an analogous affection, less severe in type, less acute in development, and not associated with undue relaxation of the bowels. A case of this kind was that of a boy, aged six years, with a neurotic father. For eighteen months he had been subject to intermittent attacks of abdominal pain, except for an interval of four or five months when he was away from home in the country. The attacks became more constant during the recent cold weather. Occasionally he had vomited. At first he had some abdominal distension, but his present condition showed absolutely nothing abnormal on physical examination. Instead of diarrhœa there was a certain amount of constipation. In such a case a diagnosis has to be based upon the history, the mode of life, and the complete absence of any apparent cause on most careful examination. Gastric and duodenal ulcers are extremely rare at this age, yet they must be borne in mind. Hydatids, gall-stones, new growths, and aneurysm of the abdominal aorta are pathological curiosities. Tuberculous adenitis or adhesions secondary to peritonitis are not infrequent. Renal colic is not unknown. Appendicular colic is an occasional cause. If such affections can be more or less definitely excluded, one is justified in a diagnosis of reflex colic, and in a good prognosis. For with accurate diagnosis and appropriate treatment the outlook is excellent. The diet clearly should consist of simple, easily-digestible food, neither hot nor cold, above all not iced, it should be taken slowly, and a certain amount of rest should be insisted on after meals. The child should be warmly clad, especially as regards the extremities. Cold and wet feet are most injurious. Of drugs, one drop of Fowler's solution before meals is the most efficacious treatment—one-half or one-third of the dose being prescribed for infants. A carminative digestive mixture is necessary if there are signs of gastric catarrh. Bromides, in addition, may do good. If, in the simple cases, the arsenic fails it may be necessary to give small doses of codeia. In the violent reflex diarrhœa of infancy, in which profuse watery stools are squirted out of the rectum as soon as food is given, from one-half to one minim of laudanum may at once stop the peristalsis, and may be given every three to six hours, if necessary, and not causing undue drowsiness.

